# Influenza A in Bovine Species: A Narrative Literature Review

**DOI:** 10.3390/v11060561

**Published:** 2019-06-17

**Authors:** Chithra C. Sreenivasan, Milton Thomas, Radhey S. Kaushik, Dan Wang, Feng Li

**Affiliations:** 1 Department of Biology and Microbiology, South Dakota State University, Brookings, SD 57007, USA; chithra.sreenivasan@sdstate.edu (C.C.S.); radhey.kaushik@sdstate.edu (R.S.K.); dan.wang@sdstate.edu (D.W.); 2 Department of Veterinary and Biomedical Sciences, South Dakota State University, Brookings, SD 57007, USA; milton.thomas@sdstate.edu; 3 BioSystems Networks and Translational Research Center (BioSNTR), Brookings, SD 57007, USA

**Keywords:** ruminants, bovine, cattle outbreaks, Influenza A, host restriction, bovine cell cultures, bovine respiratory disease, bronchopneumonia, epizootic cough, seroprevalence, MDBK cells

## Abstract

It is quite intriguing that bovines were largely unaffected by influenza A, even though most of the domesticated and wild animals/birds at the human–animal interface succumbed to infection over the past few decades. Influenza A occurs on a very infrequent basis in bovine species and hence bovines were not considered to be susceptible hosts for influenza until the emergence of influenza D. This review describes a multifaceted chronological review of literature on influenza in cattle which comprises mainly of the natural infections/outbreaks, experimental studies, and pathological and seroepidemiological aspects of influenza A that have occurred in the past. The review also sheds light on the bovine models used in vitro and in vivo for influenza-related studies over recent years. Despite a few natural cases in the mid-twentieth century and seroprevalence of human, swine, and avian influenza viruses in bovines, the evolution and host adaptation of influenza A virus (IAV) in this species suffered a serious hindrance until the novel influenza D virus (IDV) emerged recently in cattle across the world. Supposedly, certain bovine host factors, particularly some serum components and secretory proteins, were reported to have anti-influenza properties, which could be an attributing factor for the resilient nature of bovines to IAV. Further studies are needed to identify the host-specific factors contributing to the differential pathogenetic mechanisms and disease progression of IAV in bovines compared to other susceptible mammalian hosts.

## 1. Introduction

Influenza viruses belong to *Orthomyxoviridae* family and are negative-sense single-stranded RNA viruses causing acute respiratory disease in a multitude of hosts all over the world. Influenza viruses were recognized as early as the 16th century and the first pandemic officially documented was in 1580 [1]. Influenza viruses evolved to form mainly four types: alphainfluenza virus (influenza A), betainfluenza (influenza B), gammainfluenza (influenza C), and deltainfluenza (influenza D) which again diverged to subtypes and lineages, affecting multiple mammalian species worldwide, including humans. Influenza viruses undergo antigenic drift—acquiring frequent mutations in HA and NA, which enables the virions to evade the pre-existing immunity to cause seasonal epidemics/epizootics, and antigenic shift—undergoing gene reassortments causing pandemics. The most important IAV human pandemics: 1918 Spanish flu (H1N1), 1957–1958 Asian flu (H2N2), 1968 Hong Kong flu (H3N2), and 2009 swine-origin H1N1 emerged during the last century [1].

Structurally, IAV and IBV genomes have eight RNA segments, whereas ICV and IDV have only seven segments. IAV has hemagglutinin (HA), neuraminidase (NA), matrix proteins (M1, M2), and NP (ribonucleoprotein) as structural proteins; 3 subunits of the RNA polymerase complex, polymerase basic protein 1 (PB1), polymerase basic protein 2 (PB2), and polymerase acidic protein (PA); and 3 nonstructural proteins, NS1, NS2/NEP (nuclear export protein), and PB1-F2. Studies have shown that NS2 and M1 protein form complexes that can be detected in purified virions and cell lysates of virus-infected cells [2,3]. Hence, NS2 and (probably) NS1 of IAV are not considered as non-structural proteins, as these proteins can be detected in virions [4]. IBV possesses six structural proteins, HA, NA, NB, M2, M1, NP and NS2; 3 subunits of RNA polymerase complex, PA, PB1, and PB2; and nonstructural protein NS1 [5]. ICV and IDV have 4 structural proteins, M2, M1, NP, and the hemagglutinin–esterase fusion (HEF) protein that replaces the HA and NA of IAV or IBV; 3 subunits of RNA polymerase complex, P3, PB1, and PB2; and 2 nonstructural proteins, NS1 and NS2. IAV has several subtypes based on the HA and NA proteins. Currently, there are 18 HA and 11 NA subtypes, of which H1 to H16 and N1 to N9 have been isolated from birds; the subtypes H17, H18, N10, and N11 have been identified in bats [6,7]. Out of these, only three HA (H1, H2, H3) and two NA (N1, N2) subtypes have been associated with human epidemics and are capable of sustained transmission [8].

Influenza viruses spill over periodically from their primordial reservoirs (aquatic fowls) to the intermediate/secondary hosts to facilitate better adaptation and transmission and some of these hosts must remain as permanent niches for sustained IAV transmission. Other than birds, influenza A affects diverse mammalian populations such as pigs, seals, horses, dogs, cats, wild cats, minks, whales, and humans. The global pandemic of 2009 caused by swine-origin H1N1 was reported in swine, turkey, dogs, and cat [9,10,11,12,13,14]. Over the last few years, influenza infection landscape has widened to include new mammalian hosts such as bats, seals, and whales [6,15,16,17,18]. Humans are the intermediate hosts for many diseases and zoonotic infections can occur in two ways: (1) isolated, dead-end infections which fail to establish and adapt as in the case of Ebola and hantaviruses (2) virus adapts and establishes in the intermediate or secondary hosts, and also sustain horizontal transmission, as in influenza [19]. Such stable host-switch events lead to strong adaptations (ex. H5N1 and H9N2) which can resist the evolutionary pressure or the antagonistic environment posed by the novel hosts [20,21,22]. The factors that govern the virulence, pathogenicity and transmission of influenza viruses could be multifactorial including both viral as well as host factors. Host factors such as availability of the receptors, the presence of host innate immune and other cellular factors, population size and its interconnectivity all govern the sustainability of influenza transmission [23]. Influenza viral determinants undergo adaptive mutations, to expand or to limit the host range. Among the viral factors, HA glycoprotein is the primary factor determining the host range and interspecies transmission. Other viral proteins such as NP, PB2, and NS1 have also been involved in host range restriction and adaptation [24]. For example, avian influenza polymerase possesses a limited function in human hosts and hence host-specific genetic changes have occurred to the polymerase subunits and NP during natural evolution. Though uncommon in recent times, IAV has been reported in ruminant species in the past. However, a tight host genetic bottleneck might have played a major role in the evolution, preventing the adaptive mutations necessary for the sustained transmission cycles in a novel host. Interestingly, the recently emerged influenza D, for which cattle are considered to be the primary reservoir, is widespread in cattle herds across the world. In this review, we conducted a comprehensive search of the available scientific reports/journal articles on influenza over the last century, with reference to bovine species, to understand the timeline of bovine IAV incidences with respect to human pandemics and epidemics, natural and experimental infections, seroepidemiological studies, and the role of bovine cellular and host factors in the evolution of influenza.

## 2. Literature Search Strategy

**Figure 1:**
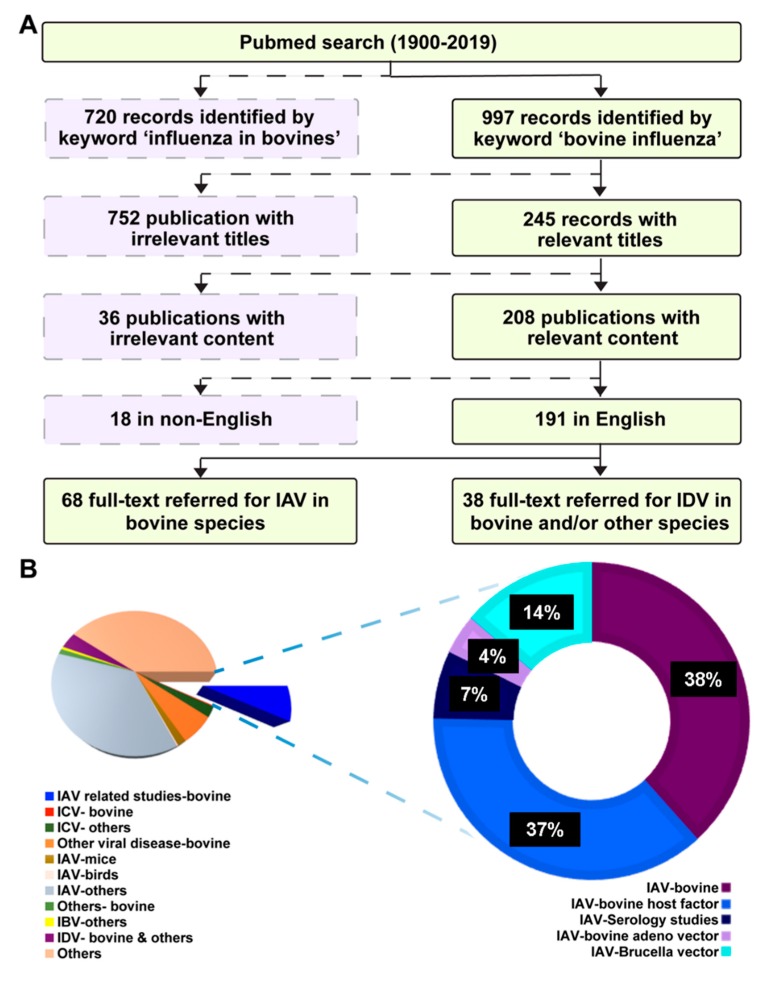
Schematic illustration of the literature search strategy. (**A**) A literature search was conducted using different keyword combinations in the PubMed database. Initial screening was carried out by shortlisting articles that matched the title for topic relevance and the availability of the articles in full text or abstract as shown in the flow chart. (**B**) Articles obtained after the keyword search were categorized based on their content as described in the pie chart. A comprehensive analyses of influenza A related studies in bovines represented by donut chart were conducted. Abbreviations used: Influenza A virus, IAV; Influenza B virus, IBV; Influenza C virus, ICV; Influenza D virus, IDV.

A literature search conducted mainly through PubMed (www.ncbi.nlm.nih.gov/entrez/query.fcgi accessed January 20, 2019) using the keywords “influenza in bovines”, “influenza in cattle”, influenza in ruminants, and “bovine influenza” yielded 720, 717, 846, and 997 results, respectively. Another search with “bovine respiratory disease and influenza” retrieved 206 results. The searches were conducted with and without specifying the dates and the results retrieved were nearly the same. For the dates specified, the search started from 1900/01/01 to 2019/01/21. A flow chart showing the schematic representation of the comprehensive search is shown in Figure 1A with dates covered from 1900/01/01 to 2019/01/21. The search yielding the highest number of results (bovine influenza, *n* = 997) was used to further categorize the journal articles in different sections. Category-wise representation of the related articles obtained by the keyword search was shown in the Pie chart in Figure 1B. Furthermore, the IAV related studies in bovine (blue pie, *n* = 73) were broken down and illustrated in a donut graph to show the different subsections (IAV studies in bovine, IAV-bovine host factor interactions, seroprevalence, and use of IAV vector for bovine diseases). There were several reports on influenza infections and respiratory diseases in cattle from Russia, Germany, and Poland, most of which were in their native languages, and hence not accessible. Few of these journal articles carried English abstracts, which have been used in this review [25,26,27]. Influenza D journal articles (*n* = 38) were reviewed separately.

## 3. A Brief Overview of Influenza Viral Ecology

Evolution and host adaptation strategies (tissue tropism, receptor binding preferences, environmental stability, and immune evasion) enable influenza virus to cross species barriers from their reservoir hosts. Furthermore, the host traits associated with the exposure (migration, and feeding behavior) and susceptibility (pre-existing immunity, species-specific host interference, age and body condition) are crucial in determining transmission efficiency and establishment of influenza in intermediate hosts. Migrating waterfowls and shorebirds have been the reservoirs of influenza viruses across the globe and almost, all influenza viruses affecting mammalian species originated from the avian gene pool [28]. Pigs are extremely susceptible to both avian and human influenza viruses because of the mixed sialic acid receptor distribution in the swine respiratory tract, consisting of both α-2,3 and α-2,6 linkages and thus are excellent mixing vessels for the different subtypes of IAV, facilitating bidirectional transmission at the pig–human interface. Swine influenza in North American pig populations, particularly in Midwest US dates to 1930 when H1N1 was initially isolated in pigs [29]. This classic lineage derived from 1918 Spanish pandemic flu, is a robust virus and continues to circulate in the swine and human populations. Besides, H3N2 avian–mammalian reassortant viruses [30,31], numerous variants of second generation reassortants between human pandemic H1N1 2009 and pig endemic H1N1 viruses, and H1N2 viruses have evolved over time and became well-established in swine populations [32]. IAV subtype distribution in equine populations included H7N7 and H3N8. However, since the 1970s, H3N8 has been the predominant subtype circulating all over the world. Few exceptions were the avian-like A/equine/Jilin/1/89 (H3N8) which was antigenically different from the H3N8 viruses isolated between 1963 and 1991 [33] and H5N1 isolation from a horse in Egypt [34]. Dogs were affected by equine H3N8 and H3N2 [35,36] viruses, whereas cats were infected by avian influenza viruses, mostly H5N1 subtype [28]. Aquatic mammals such as seals also harbored influenza A viruses in the early 1980s, particularly the H7N7 and H4N5, associated with low mortality and later, with H10N7 which caused massive deaths [17,37]. IAV subtypes H13N2 and H13N9 were isolated from the lungs and hilar nodes of the pilot whale [16]. Minks were affected by both mammalian and avian origin influenza viruses such as H10N4, H10N7, H9N2, H5N1, swine variant H3N2, H1N2, and H1N1 pdm 2009 [38,39,40,41,42,43,44].

**Figure 2:**
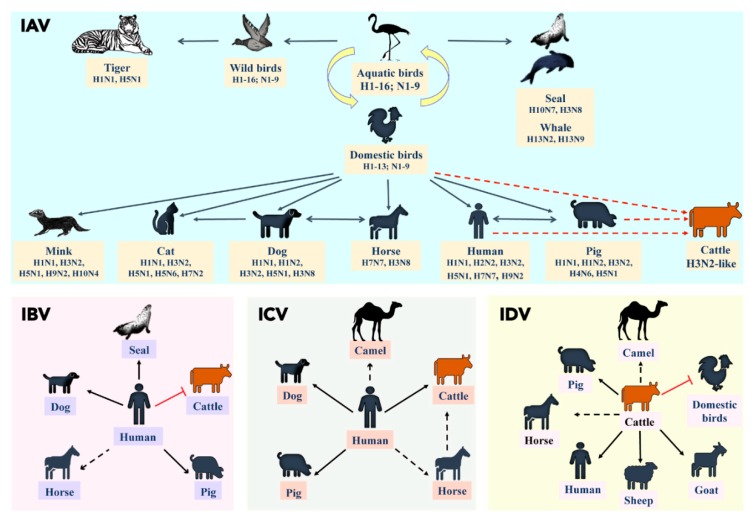
The host range for all four types (IAV, IBV, ICV, and IDV) of influenza viruses. The association of influenza disease in different mammalian species with reference to cattle (orange) is described separately. In each box, the bold black line represents active infection/disease; dotted black line represents the exposure without active infection/disease, as demonstrated by the serological evidence. A red dotted line in IAV indicates the possible source/routes of IAV infection (human, avian or swine) that can occur in bovines. Red blocked line indicates neither disease nor exposure. Vector graphic images used in the figure were taken from icon pool of the Microsoft Office and Freepik (www.freepik.com).

A schematic diagram illustrating the potential mammalian hosts for the four types (IAV, IBV, ICV, and IDV) of influenza were given in Figure 2. Previous studies have reported that cattle influenza A isolates were found to be either human IAV or related to human IAV strains, especially the HA glycoprotein was similar to the prototypic human H2 and H3 subtypes [45,46]. Furthermore, swine IAV strain has also been isolated from cattle in Hungary, which indicated that source of infection could be human, swine or poultry as shown by the red dotted lines in Figure 2 [47]. However, all the reported cattle-origin IAV strains originated during 1971–1974, and very few studies were available using these cattle-origin strains [27,48]. Taken together, the subtype distribution of influenza A over the last 45 years clearly indicated that influenza A viruses established its niche and has evolved in almost all mammalian hosts at the human–animal interface, except in bovine species.

While IAV evolved in multiple mammalian species, IBV and ICV primarily infected humans (Figure 2). IBV has no subtypes but diverged into two major lineages causing localized epidemics affecting humans and seals [49,50,51]. The susceptibility of domestic piglets to influenza B virus has been reported as early as in 1966 [52]. Furthermore, serological screening of Midwest US swine herds from 2010–2012 demonstrated that 38.5% (20/52) of sampled farms and 7.3% (41/560) of tested swine sera were positive to IBV [53]. Antibodies against IBV were also reported in dogs and horses [54,55,56]. Further studies are needed to investigate whether IBV affects other mammalian hosts and till date, IBV was not reported in ruminants except for a seroprevalence reported in the USA (B/Johannesburg/59) and in Italy (B/Bonn and B/Roma/1/59) [47].

Compared to IAV and IBV, ICV infections caused mild disease and were found to have coexisted with IAV and IBV in humans [57]. Serological screening in Japan and Great Britain revealed ICV incidences in pigs in the 1990s [58,59]. Natural infection of ICV in pigs was also reported from China in 1981 [60]. Serological evidence of ICV was reported in Midwest equine populations of the USA [61]. Surprisingly, ICV was also identified in the respiratory samples of cattle from Western Canada and North America and complete genome of bovine ICV has been isolated recently from one of the respiratory samples collected from 2016–18 in North America [62,63]. While the transmission of ICV from human to pigs/horses/cattle or vice versa is still under debate (Figure 2), further investigation is warranted to determine its host range, virus tropism, and transmission potential.

Recently emerged “type D influenza” virus (IDV) demonstrated 50% overall amino acid identity to ICV and displays distinct differences from influenza A and B viruses. Influenza D virus was originally isolated from a piglet showing influenza-like symptoms in Oklahoma in 2011; however, IDV is more widespread in cattle herds than pigs in North America and parts of Eurasia [64,65,66,67,68]. Bovines were considered to be the primary reservoir for IDV with periodic spill over to other mammalian hosts such as pigs, horses, camels and small ruminants [61,69,70,71]. High seroprevalence of IDV was reported in occupational workers and recently IDV genome was detected from a respiratory sample of a pig farm worker, and also from bioaerosol samples derived from high traffic human environments such as emergency hospital rooms and airports, which implicate its public health significance [71,72,73]. Collectively, the comprehensive analyses of the host range of four influenza types suggested that cattle are the primary hosts for IDV and could be susceptible to the other three influenza types (Figure 2).

## 4. Role of Influenza in Bovine Respiratory Diseases and Evidence of Zoonosis/Reverse Zoonosis

Since time immemorial, domestic cattle are the backbone of the food and agriculture and their roles remained equally significant in the modern world. However, cattle distribution and the production and consumption of milk and meat are uneven across the different countries of the world. Among livestock, pigs have been the mixing vessels for avian and human influenza A viruses and the association of humans with swine facilitated the bidirectional transmission of influenza at the pig–human interface. Interestingly, cattle have been domesticated by humans 10,500 years ago [74], whereas pigs were domesticated 9000 years ago [75]. Moreover, the close association of humans with cattle should naturally make them vulnerable to bidirectional influenza transmission, which is not the case. This raises numerous questions for which the answers are not clearly known yet. Are cattle susceptible to influenza A viruses? Despite the mutational robustness, rapid evolution, and diverse host ecology of the influenza A virus, why ruminants were spared from the infection? What happened to the cattle associated influenza A viruses emerged during the early 1970s? Whether or not the virus surpassed/escaped species-specific host immune selection pressure? The purpose of this review is to seek answers to these questions by performing comprehensive analyses on the past influenza incidences occurred in ruminants.

The first report on influenza disease in bovines dated to 1949 from Japan; however, no cattle-origin IAV was isolated from these outbreaks [76]. Concurrent to human pandemics, cattle also exhibited influenza-like respiratory disease and several cattle influenza A strains were isolated from different parts of Europe and Russia around this time. A few of these strains had HA and NA glycoproteins similar to prototypic human H2 and H3 subtypes, while others were dissimilar to human IAV [46,47]. It could be believed that IAV transmission from humans to cattle occurred in the past. However, these cattle influenza A viruses failed to adapt and establish their niche for a sustained transmission and evolution due to stringent transmission bottlenecks.

The notable respiratory tract infections of cattle in the past emerged during 1950–1970 which included bovine parainfluenza 3 (BPIV3), bovine herpesviruses (BHV) such as infectious bovine rhinotracheitis virus (IBR), bovine viral diarrhea virus (BVDV), and bovine respiratory syncytial virus (BRSV) [77,78,79,80,81,82,83]. These viral etiologies coupled with the secondary bacterial infections constituted bovine respiratory disease complex, a highly significant respiratory disease condition causing high morbidity in cattle and huge economic loss to the cattle farmers in North America [84]. Bovine respiratory disease (BRD) is a multifactorial disease caused due to a single or combination of factors such as environmental, host, managemental, and infectious factors, posing significant economic burden affecting the cattle industry in North America. The morbidity rate of BRD is about 70–80% in the USA, amounting to a loss of $23.60 per morbid calf [84]. Other than BVDV, BHV, BRSV, BPIV3 against which commercial multivalent vaccines were well developed, metagenome sequencing of the respiratory samples from cattle identified other viral agents such as bovine rhinitis viruses, bovine reovirus, bovine corona (BCV) and enteroviruses [85].

Metagenomic virome sequencing in symptomatic and asymptomatic feedlot cattle detected BHV1, BVDV, BRSV, BCV, and IDV in symptomatic animals at a rate of 2.1, 8.5, 2.1, 29.8 and 17.0% respectively and found IDV as a moderate risk factor in BRD complex (*p* < 0.15) [85]. Even though a low seroprevalence (0.6%) of influenza C in bovines was reported around the late 1970s [56], influenza C prevalence in cattle or any other ruminants was not reported elsewhere until 2016. Recently, ICV has been detected in 64/1525 (4.2%) of bovine respiratory tract samples (nasal swabs and lung specimens) collected from animals suffering from acute respiratory disease during October 2016–January 2018 by RT-qPCR [62]. Furthermore, sequencing of 590 base pair (bp) fragment of the matrix gene from 12 ICV-positive samples revealed 98% sequence identity between strains and 95% identity to the human ICV strains, which clearly indicates the bidirectional transmission of ICV at the human–cattle interface and reverse zoonotic potential [62]. Complete genome sequencing of one of the positive ICV isolates has been performed and designated as C/bovine/Montana/12/2016 [86]. Within the 12 ICV strong positive samples, four samples also harbored IDV. Interestingly, metagenome sequencing of the paired nasal swab and tracheal washes from the Western Canadian beef cattle also detected influenza D associated with BRD, however, influenza C was detected from a cattle without respiratory disease [63,87]. While it is largely unknown whether ICV and IDV could undergo reassortment in bovines to cause disease and facilitate cross-species transmission, the IDV involvement in the BRD complex has been established beyond doubt. The recent developments in IDV infection landscape such as serological evidence, and the recent detection of IDV genome in occupational workers coupled with presence of IDV in the bioaerosol samples of the high traffic human environments such as airport and hospital emergency room [71,72,73] indicate the possibility of a sustained influenza D transmission at the cattle–human interface. Taken together, these findings suggest that interspecies transmission could occur between cattle and humans and involves a potential risk of zoonosis/reverse zoonosis.

## 5. Natural Cases of Influenza A in Bovines

First recorded evidence of influenza in cattle occurred in 1949, where 160,000 cattle were infected in the western and middle part of Japan [76]. This incidence of cattle influenza ran for a short course with recovery in 2–3 days and the documented symptoms included high temperature (40–42 °C), blepharitis, nasal discharge, anorexia, tympanites, pneumonia, joint problems, and a decrease in lactation. This report also mentioned about some major cattle influenza outbreaks occurred previously in the Fall of 1889 and 1893, and some minor outbreaks in 1914–1916 in Japan [76]. The same study also mentioned an experimental infection of 11 calves, using nasal discharge/defibrinated blood from diseased animals and the successful virus isolation in mice, characterized by few deaths and lung and liver lesions at the 20th serial passage.

The first report on influenza virus isolation from animals was documented by Romvary et al. [88] from Hungary in 1962, which described the isolation of IAV strains similar to human H2 HA glycoprotein from pigs and sheep during 1959–1960. Romvary et al. [46] also isolated porcine IAV strains bearing human H3 HA glycoprotein. Lopez and Woods reviewed influenza viruses from cattle and the first cattle-origin influenza isolate was reported by Barb et al., 1962, cited in [47]. Furthermore, there were reports on cattle influenza from several countries primarily from the old Union of Soviet Socialist Republics (USSR), and the publications were mostly in the Russian language, with the rare occurrence of English abstract and keywords. Among these, the earliest report was on the seroepidemiological study of influenza in domestic species of animals in 1969 [25]. During the period 1970–80, cattle isolates of influenza A have been reported from different parts of the world, post/around the time 1968 Hong Kong H3N2 pandemic occurred in humans. In 1973, the isolation and identification of the A/Hong Kong/1/1968 (H3N2) virus from cattle suffering respiratory diseases were reported in Russia [45]. The earliest cattle influenza A strain studied under experimental conditions was A/calf/Duschambe/55/71 (H3N2) from Russia. This strain was derived from a natural case of respiratory illness in a terminally ailing calf and was isolated in embryonated chicken eggs [45]. Both H1N1 and H3N2 strains were isolated from cattle later. Few of these isolated strains reported include Sw/Shope (H1N1) from Hungary and several H3N2 strains from the USSR. The two viruses isolated from Hungary and the USSR possessed type 2 neuraminidase; however, HA glycoproteins were unidentified [47]. The H3N2 strains were similar to the prototypic human H3N2 strain A/Hong Kong/1/1968 (Schild G, C., World Influenza Center, London). Furthermore, studies on influenza outbreaks in cattle occurred in Russia in 1973, and antigenic characteristics of the influenza viruses isolated from the birds and animals of the USSR were reported, both of which were in non-English languages [89,90]. Another study on the isolation of IAV strains from nasal swabs and lung samples of acutely ill cattle were reported from Budapest, Hungary in 1974 [46] characterized by mortality and the disease lasted about 1–3 weeks. The affected animals included calves (4–6 months of age), young, and beef cattle, who suffered epizootic cough associated with respiratory symptoms such as nasal discharge, lacrimation, anorexia, and lethargy. Gross lesions involved focal acute catarrhal pneumonia, alveolar and interstitial emphysema, bronchitis with hemorrhages due to asphyxia and no extrapulmonary lesions. Interestingly, there was no bacterial involvement reported in this study and the virus isolation using embryonated chicken eggs resulted in the death of the embryos at 4th day [46]. Furthermore, in 1976 and 1977, there were studies from Russia, describing cattle influenza [91,92]. In 1978, Wagner et al. [93] described influenza and enzootic bronchopneumonia in cattle from Germany. In Germany, the reports on examination of cattle for influenza and pathological examination of the lungs from cases of bovine influenza were available as early as 1977 [91,94]. However, no further reports on influenza-related respiratory/non-respiratory diseases in bovine/any ruminant species were available until the late 20th century.

In 1997, an idiopathic condition manifested in dairy cows in Bristol, southwest England with a sporadic drop in milk production [95]. Brown et al. [96,97] also reported seroconversion against influenza A in cattle from Great Britain, which was markedly associated with reduced milk yield and respiratory disease. However, the virus isolation from these seroconverted animals was unsuccessful. Interestingly, these cattle seroconverted to influenza A virus alone, with no detectable antibodies against BVD, IBR, PI3, and BRSV, suggestive of the etiological role of influenza A in the reduction of milk yield. Furthermore, in 1999, Gunning et al. [98] also reported that the natural cases of influenza in milking cows increased with an annual incidence rate of 10–20% in some herds of England with a sudden drop in milk yield, mild pyrexia, anorexia, occasional respiratory signs such as nasal discharge and increased respiratory rate. High levels of neutrophils and haptoglobin were present in the blood in most of these cases. Serological screening of paired sera collected from five cattle herds with the same clinical history against IBR, PI3, BRSV, adenovirus, *M. bovis*, *H. somnus*, *C. psittaci*, *C. brunetti*, *P. hemolytica*, *P. trehalosi*, treponemes revealed antibodies against BRSV and PI3 in all herds, while BVD and IBR were detected only in some herds. On the other hand, these cattle sera demonstrated significantly high antibody titer to two human IAVs: 60% for A/England/333/80 (H1N1) and 65% for A/England/427/88 (H3N2) and only 5% of the cows were seronegative against both viruses [98]. These observations clearly indicated the exposure and natural susceptibility of cattle to human influenza A viruses.

Concurrently, Dr Ian Brown and his colleagues at Veterinary Laboratories Agency near Weybridge, United Kingdom reported the presence of influenza genes in cattle around the late 1990s (https://www.nature.com/news/1998/020107/full/news020107-4.html. accessed January 21, 2019). However, no related peer-reviewed records were available. In Northern Ireland, a seroepidemiological study conducted on 84 paired acute and convalescent cattle sera collected from 17 outbreaks, against A/England/333/80 (HIN1) and A/England/427/88 (H3N2) during 1998–1999, with clinical manifestations of respiratory disease, diarrhea, and milk drop syndrome demonstrated seroconversion in 56.5 and 58.8% of the convalescent sera against H1N1 and H3N2 respectively. While H3N2 antibody titers were higher compared to H1N1 in general, this study also revealed a higher rate of seropositivity against human H3N2 over porcine H3N2 strains. However, virus isolation in specific pathogen-free chicken embryos was unsuccessful from 142 cattle with similar clinical manifestations [99]. The association of human influenza A viruses with milk drop in cows was prevalent in the early 2000s. In 2008, Crawshaw et al. [100] demonstrated rising antibody titers against same human influenza viruses, A/England/333/80 (H1N1) and A/England/427/88 (H3N2) from a Holstein Friesian herd suffering from acute fall in milk production and tested seronegative against BRSV, BVD, IBR, and PI3 viruses. The loss of milk production (difference in the mean milk production between the uninfected animals and affected animals) was measured to be 159.9 L, which accounted for 2% of the lactation yield per cow. Analyses of the clinical data revealed pyrexia and increased respiratory noise in the high seropositive animals with no detectable difference in respiratory rate. The authors of this study also discussed an unpublished data on contemporary evidence of influenza A in cattle, detected by RT-PCR in a lung tissue sample from a calf suffering from pneumonia [100]. However, another study conducted to find the infectious etiology of the fatal cases of calf pneumonia in 48 calves from 27 herds around this time, did not detect influenza A virus. On the contrary, this study demonstrated that severe lung pathology in calves is due to non-influenza factors such as Mannheimia–Pasteurella in 36/40 (90%) cases; *Arcanobacterium pyogenes* in 16/40 (40%) cases; *Mycoplasma bovis* in 12/40 (30%) cases; bovine respiratory syncytial virus in 4/40 (10%) cases; *Histophilus somni* in 2/40 (5%) cases, while bovine herpesvirus-1, bovine viral diarrhea virus, and parainfluenza virus-3 were detected in 1/40 (2.5%) cases [101]. Taken together, human-like influenza A viruses circulated in cattle at two different time frames: 1970–1981, and 1997–2006 in different parts of the world, clearly indicating that cross-species transmission occurred at the human–cattle interface. Relevant references pertaining to the natural infections or outbreaks of cattle IAV were listed in chronological order in Table 1.

**Figure 3:**
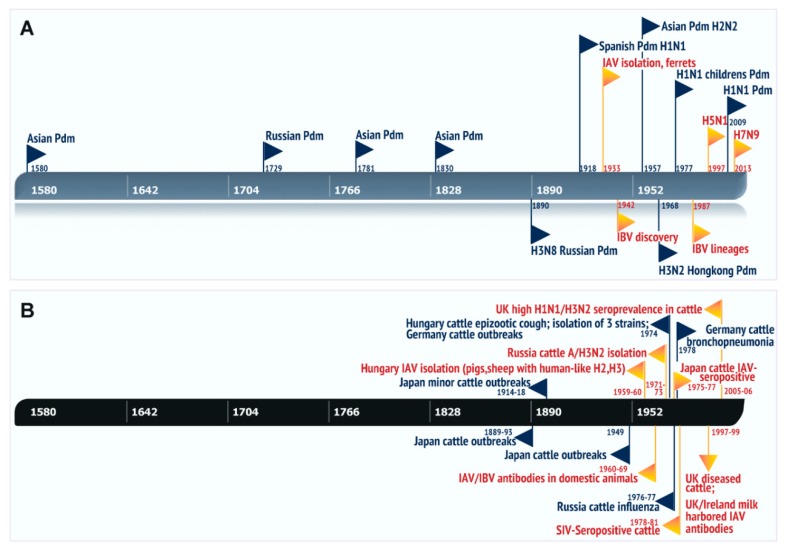
History timeline of the major influenza events in the past (**A**) Known human influenza pandemics along with the important outbreaks/discoveries. All the pandemics with year and country of origin are indicated in “black” flag, black font, while the other outbreaks/epidemics/discoveries are indicated in “orange” flag, red font. (**B**) influenza A incidences occurred in ruminants especially bovine species, were plotted based on the information from peer-reviewed articles. Only the natural infections/outbreaks and seroepidemiological studies reported in cattle in the past were included in the timeline. Experimental studies were excluded in this timeline. Illustrated major events included the major influenza outbreaks, cattle epizootics/respiratory disease with year and country of origin, and are represented by black triangle blocks, black font. Please note that both the timelines (A and B) have same dimensions starting with human pandemic as early as 1580 to 2013 H7N9 outbreaks and the timeframe of these cattle outbreaks/diseases (1889, 1893, 1914–1916, 1949, 1959–1960, 1971–1981, 1997–1999, 2005–2006) occurred almost concurrent to the human pandemic/outbreaks, which is indicative of the natural susceptibility of bovines to human influenza viruses. Most of the cattle influenza A isolates appeared to be related to human influenza A strains, with the HA glycoprotein similar to the prototypic human H2 and H3 subtypes.

A timeline of all the reported cattle epizootics and respiratory diseases with influenza viral etiology plotted in relation to the known human influenza pandemics is illustrated in Figure 3 [1,45,46,47,48,56,76,88,90,92,93,95,96,97,100,108,109,110,111,112,113,114]. The sequence of events indicated that most of the influenza-related outbreaks in cattle with clinical manifestations such as bronchopneumonia, epizootic cough, respiratory distress, and sporadic milk drop have occurred, almost concurrently with some of the human pandemics (Figure 3). Sporadic milk drop in cows reported in different parts of Europe demonstrated a strong causal association with significant rising antibody titers against human H1N1 and H3N2 viruses [100]. The fact that a few cattle IAV strains isolated around the 1970s were related to the prototypic H2 and H3 subtypes of human IAV and the presence of IAV and IBV antibodies in domestic animals indicates that human influenza-like viruses circulated in cattle and other domesticated animals in the past.

## 6. Experimental Infections of Influenza A in Ruminant Species

Several experimental studies have been performed in the past to study the antibody responses in different species of domestic animals. In 1956, an experimental infection by direct inoculation of influenza A PR8 strain and Newcastle disease virus (NDV) into the lactiferous sinus of goat mammary glands resulted in the production of neutralizing antibodies in the milk and blood. This study also found that influenza neutralizing antibody level in the blood phased out slowly compared to the NDV even after surgical removal of the mammary gland [115]. In 1974, experimental inoculation of seronegative yak with human IAV such as A/Hong Kong/1/68 (H3N2), A/England/42/72 (H3N2), and A/equine/Prague/1/56 resulted in serological response against all these strains [103]. Experimental inoculation of calves with A/csf/Udmurtiia/116/73 was reported in 1977 [27]. Nakamura et al. [110] experimentally infected calves with influenza B virus by aerosol and intravenous routes to determine the serum non-specific inhibitors of influenza in domestic animals. Several experimental studies have been reported from cattle in the 20th century, and some of the reports were only available in non-English languages [25,26,27]. Experimental infection in different mammalian species such as calves and lambs to characterize the antibody responses revealed appreciable hemagglutination inhibition (HI) antibody titers in calves after primary infection, leading to an anamnestic response after challenge with a homologous antigen [110].

Experimental infection using human and swine IAV subtypes caused variable responses in causing respiratory disease in cattle. In 1977, calves experimentally infected with three Hong Kong-like H3N2 strains (A/Michigan/l/72, A/England/42/72, and A/Aichi/2/68) compared to the calf H3N2 strain, A/calf/Duschambe/55/71 demonstrated no respiratory disease by human IAV, while A/calf/Duschambe/55/71 caused disease with nasal discharge, cough, and mild rhinitis. However, virus shedding was detected for A/Aichi/2/68 and A/calf/Duschambe/55/71 for five and seven days respectively, indicating that calves were susceptible to human IAV. The A/calf/Duschambe/55/71 strain was considered to be a host range variant of Hong Kong/68 strains isolated from humans [48]. Furthermore, intranasal inoculation of live swine influenza virus (SIV) A/sw/IL/75 (H1N1) in calves caused respiratory disease with virus shedding, contact transmission to sentinel animals, seroconversion at 9 days post-infection (dpi) and virus neutralization antibody development at 14 and 21 dpi [105]. Another study using recombinant vaccinia virus expressing SIV-HA (A/NJ/11/76) developed antibodies when inoculated to cattle, sheep, and poultry, while the wild-type virus did not cause any antibody response. No contact transmission was reported by the wild-type or recombinant virus in these species [104].

The pathogenesis and transmission of avian influenza viruses in bovine species have also been studied previously. Cattle egrets share a symbiotic relationship with cattle and forages along with cattle and other livestock species. Recently, highly pathogenic avian influenza virus (HPAIV) was isolated from cattle egret in Egypt near broiler chicken farms [116]. Therefore, it would be noteworthy to study the epidemiological role of the cattle egrets in the transmission of IAV. Cattle egrets were intranasally challenged with an HPAIV (A/duck/Vietnam/40D/04 (H5N1)) to study its role in H5N1 outbreaks in Vietnam and to investigate the virulence of the strain. The egrets contracted the infection, and some succumbed to death in a week. However, no contact transmission occurred in the co-housed chickens [107]. Despite the close association of cattle egrets or similar intermediate hosts of influenza, it is interesting that cattle are refractory to influenza. In 2007, experimental inoculation of calves with HPAIV strain, A/cat/Germany/R606/2006 (H5N1) demonstrated 100% seroconversion with neutralizing antibodies against the homologous strain. This study reported very low viral shedding as determined by the titration of nasal swab fluid in embryonated chicken eggs and MDCK cells. Virus neutralization and the ELISA tests conducted at 3 months post inoculation demonstrated seroconversion in all the inoculated calves and one contact animal, thus providing evidence for contact transmission [106]. On the contrary, another experimental infection of six beef calves and two ponies with A/equine/Kentucky/91 (H3N8) did not cause any viral shedding, clinical symptoms, or disease in calves compared to the ponies [117]. Table 1. describes the experimental infection conducted in cattle in chronological order. These experimental studies show that ruminants could be infected with IAV derived from avian, swine, and human IAV subtypes.

## 7. Seroprevalence Studies of Influenza A in Bovine Species

Influenza serosurveillance studies have been conducted in bovines and other animal species over time in different parts of Eurasia. In Romania, epidemiological studies conducted in humans and animals during the early 1960s by Bronitki et al. [110,118] demonstrated influenza A and B specific antibodies in several wild and domestic animals including cattle, and sheep. In the early 1970s, naturally occurring antibodies against the H3N2 viruses were detected in the crossbred yak, cattle and water buffaloes in Kathmandu, (Nepal) and goats and cattle in West Bengal (India) as demonstrated by the single radial immunodiffusion tests [103]. The precipitation and complement fixation tests to study the incidence of the A/Port Chalmers/73 (H3N2), A2/Hong Kong/1/68 and PR8 in fourteen animal species from Ottawa area in Canada revealed seropositivity in six species such as dog, cat, rabbit, goat, chipmunk, and sheep [119]. In Great Britain, a serosurveillance study conducted on swine and bovine samples collected during September 1973–July 1977, against A/Swine/Wisconsin/66 (HSwlN1), A/Swine/1976/30 (HSwlN1), A/Hong Kong/1/68 (human H3N2), A/Port Chalmers/73 (human H3N2) demonstrated seropositive swine samples against human H3N2 viruses with 4.5% (1974), 1.7% (1975) and 2.3% (1976) against A/Port Chalmers/73 suggesting the evidence for reverse zoonosis. On the contrary, none of the bovine sera was positive against the tested swine (H1N1) and human (H3N2) influenza viruses [120]. Seroprevalence study conducted in different species of animals involving cattle, horses, pigs, dogs, cats, minks, and rats over the period 1975–77 in Japan, demonstrated antibodies against 15 subtypes of influenza A viruses among 16 subtypes that were tested [111]. In the case of cattle, out of 728 serum samples, only 1.5% and 1% were seropositive against H0 and H3 Aichi respectively and were seronegative against other subtypes [111]. In 1978, a seroprevalence study of influenza B and C viruses in different mammalian species including cattle conducted in Japan revealed that all cattle sera were negative to IBV and only 0.6% seropositive samples against ICV [56].

In Northern Ireland, around 200 sera collected from crossbred and indigenous sheep breeds to study the seroprevalence of influenza A and other viruses (parainfluenza types 1, 2, and 3, respiratory syncytial virus, bovine adenovirus, Maedi-visna virus, and bovine viral diarrhea virus) demonstrated no antibodies against influenza A, Maedi-visna and parainfluenza virus 1 and 2 [121]. A review of the published pathological and serological studies by Lopez and Woods, reported seroprevalence of influenza A and B viruses (complement fixation and hemagglutination inhibition tests) in ruminants from different parts of the world such as the USA, Italy, Rumania, USSR, Nepal, India, and Hungary [47,122].

Another serological study conducted in five calves inoculated with swine influenza virus showed a significant association between the mean diameter of the hemolysis zone obtained by the single radial hemolysis (SRH) test and the geometric mean of the HI titer after periodate treatment and receptor-destroying enzyme [123]. In 1974, serological screening against human influenza viruses such as A/Hong Kong/1/68 (H3N2), and A/England/42/72 (H3N2), conducted in West Bengal, India (cattle, goats) and Kathmandu, Nepal (water buffaloes, cattle) also showed seropositivity against the H3 antigens by single radial immunodiffusion test [103]. While this study demonstrated seroprevalence against H3 antigens, no antibodies were detected against the equine influenza A virus, A/equine/Prague/1/56 (Heq1 Neq1) in human, goat, cattle, chicken, and dog sera [103]. Another serological survey involving 177 paired calf sera from 1978–1981 showed that 3.4% of the calves were seropositive to swine influenza virus [122]. A retrospective serological survey conducted in Minnesota, USA involving 2,345 bovine sera against H1 subtype-specific antigen revealed 27% positive and 31% low positive samples, without any clear evidence of clinical infections [114]. This study also found that peak titers occurred during September–November, and February–March [114]. In Egypt, seroprevalence against avian influenza has been demonstrated in samples collected from different animal species such as goat, cattle, buffaloes, sheep, horses, swine, donkey, sewage rats, stray dogs, and cats [124]. Bovine seroepidemiological studies on the prevalence of equine and porcine influenza viruses in cattle sera collected during 1999–2000 in Kentucky demonstrated 17% and 51% seropositive samples against A/equine/Kentucky/94 (H3N8) and A/swine/Texas/98 (H3N2) respectively [117]. A chronological list of studies pertaining to the seroprevalence of IAV in ruminants is shown in Table 2. These observations have clearly indicated that bovines are susceptible to human, avian, equine, and swine influenza viruses; however, there appears to be some host-specific interference preventing disease development.

## 8. Bovine Cell Cultures for Influenza Studies in Vitro

Bovine-derived cell cultures especially Madin–Darby bovine kidney (MDBK) cells were widely used for influenza research and these cells played an important role in the phenotypical and genotypical studies of the influenza virus then and now. During 1940–1970, the nucleic acid structure of influenza virus was widely studied, and the virus cultures were mainly derived from infectious allantoic fluid, chorioallantoic membranes, and fibroblasts of the chicken embryos [126,127,128,129,130,131,132,133,134,135,136,137,138,139,140,141,142]. Various studies on the structure and segmented genome of influenza have been demonstrated as early as 1962 [143]. Bovine cell cultures have been widely used for the antigenicity and pathogenicity studies of influenza A and B types. The first report on propagating high yield infectious influenza virus was carried out by Choppin, P. W. in 1969 using MDBK cells, a continuous cell line derived from bovine kidney [144]. Since then, MDBK cells have been used to grow the virus stocks of influenza, especially A/WSN/1933(H1N1) strain (WSN) to study the structure and assembly [145,146,147,148]. WSN-infected MDBK cells have been used for the quantitative measurement of the plus-strand and minus-strand RNAs synthesized during the early and late replication cycle [149]. Purified virions of influenza A, WSN strain grown on MDBK cells were separated by gradient centrifugation and electrophoretically analyzed to determine seven polypeptides [150,151]. The isolation of ribonuclease protein (RNP) with RNA polymerase activity by discontinuous sucrose gradient fractionation of influenza-infected cells using the BHK-21F cells and MDBK cells was also described [152]. MDBK cells have been used in characterization and structural studies of influenza [153,154,155]. The calf and human fetal kidney cells were also used to demonstrate the plaque formation by influenza B viruses [156]. An improved virus propagation system based on the roller cultures has been demonstrated using bovine embryo kidney cells and MDBK cells [157,158]. Simultaneously, pig and canine kidney continuous cell lines were also used to study the persistent infection of influenza and to study the nuclear protein and two nonstructural proteins NS1 and NS2 [159,160,161].

Inoculation of influenza virus on MDBK cells caused the production of M and NS gene segments in full length, without virus production, when infected by a mutant A/WSN virus for several passages, indicative of defective genomes [162]. The concept of Von Magnus particles and defective interfering (DI) influenza viruses was studied using MDBK and HeLa cells and found that DI viruses produced during persistent infection were in good correlation with the ability of the host cell species to produce the infectious virions [163,164,165]. Host-dependent variation in the relative amount of the cleaved and uncleaved HA polypeptide during influenza infection was reported, by comparing the amount of HA by the WSN strain of influenza A0 and the RI/5-strain of influenza A2 in primary monkey kidney cells, MDBK, BHK21-F, and chicken embryos. It was also found that uncleaved HA occurred more in the early growth cycle of influenza virus than the late stage when the cytopathic effects were more pronounced [166]. HA polypeptide cleavage by plasmin produced by the host cell plasminogen activators was also studied in MDBK cells [167]. Plaque formation by influenza type A and B viruses has been extensively studied in calf kidney and specific pathogen-free chicken kidney cells and found the linear relationship of plaque number and virus concentration [168].

MDBK cells have been widely used to study the biological properties of mutant influenza viruses, produced by the host cell-mediated selection pressure. Influenza A, WSN strain caused fuzzy plaques on the chicken embryo fibroblasts but clear plaque morphology when grown in MDBK cells. The parental fuzzy virus and mutant clear viruses demonstrated different binding affinities, with clear viruses producing a high yield, with large amounts of mRNA, exhibited hemagglutination property in the presence of calf serum components and remained cell associated when transferred from 0–37 °C, unlike the fuzzy viruses [169]. Another interesting feature about the influenza viruses derived from different host cell systems is about the amount of the carbohydrate added to the viral HA protein, which in turn determines the host binding property. A study conducted using WSN-F strain of influenza A in chicken embryo fibroblasts and MDBK cells in the presence of tunicamycin showed that HA generated by the MDBK cells, contained 4000 Daltons of carbohydrate in excess than the virus grown in the chicken embryo fibroblasts, which reduced the receptor binding affinity of the virus. This study also demonstrated that MDBK derived influenza viruses have more highly branched and complex asparagine-linked oligosaccharides and galactose-containing bisected oligosaccharides compared to HA subunits derived from viruses grown in chicken embryo fibroblasts [170]. The role of host cell-specific glycosylation of the HA1 subunits, particularly at residues 129 (located at the tip of the HA) and 184 (close to the receptor binding pocket), in receptor binding properties of three influenza variants was studied in MDBK cells. This study demonstrated that glycosylation is site-specific in all the virus variants that were grown in MDBK cells and the reduction in the receptor binding properties of influenza viruses associated with MDBK cells is due to the cumulative effect of the presence of large complex glycans at position 129 and His to Asn substitution at residue 184 [171]. The glycosylation pattern of HA is dependent on the host cell origin which determines the size of the HA, its receptor binding properties, interaction with neutralization antibodies [172]. Further studies are needed to understand whether this peculiar pattern of HA glycosylation has any role in restricting the influenza pathogenesis in bovines in vivo.

The utility of primary bovine kidney cells for developing recombinant temperature-sensitive mutants for human immunization was reported in 1972. The recombinants were developed by co-infecting primary bovine kidney cells with wild-type influenza A/ Hong Kong/1968 (H3N2) and influenza A/Great Lakes/1965 (H2N2) viruses [173]. Several other studies using temperature-sensitive mutants of influenza also used MDBK cells for virus propagation [174,175]. Tracheal/lung organ cultures derived from cow and pig embryos were employed for influenza A2 virus propagation and demonstrated that tracheal organ cultures were more sensitive and supported virus replication [176]. Bovine and porcine tracheae have been used as explant cultures to study the interaction between Mycoplasma and influenza, where trachea of both animal origin was infected with *Mycoplasma hyorhinis* and superinfected with influenza and vice versa. The swine trachea showed a synergistic pathologic effect with a complete loss of the ciliated epithelium when infected with *Mycoplasma hyorhinis* on day 0 and superinfected with swine influenza on day 2 of the study while the bovine trachea did not show such pathology under similar conditions. On the other hand, swine trachea infected with influenza and superinfected with Mycoplasma clearly demonstrated enhancement of the Mycoplasma growth compared to the controls [177]. Cow embryo tracheal organ cultures and kidney tissue cultures were used to study the reactogenic and immunogenic changes of influenza A/Hong Kong/1/68 (H3N2) virus over serial passages [178]. Virus propagation of WSN influenza strain in bovine, human and chicken embryo cell culture demonstrated that the airborne stability of the WSN virus varied between cell culture versus embryonic eggs and the maximum stability was obtained at low relative humidity [179]. Bovine nasal turbinate cells were also used to study the viral replication kinetics of equine influenza virus (EIV), H3N8 [117,180] and found that EIV infected cells showed cytopathic effect characterized by cell shrinkage rounding and detachment from the cell matrix, compared to the mock cells at 48 h post infection. However, experimental infection of EIV H3N8 virus in calves did not cause any disease or virus shedding despite the estimated equine influenza seroprevalence of 17% in cattle sera collected during 1999–2000 [117]. These observations where in vitro bovine cell/organ cultures can support influenza viruses while in vivo conditions were more resistant, also suggest that bovines possess some host/biological entities interfering with the influenza viral replication and affecting the pathogenesis. A chronological list of references about the utility of bovine cell/organ cultures for influenza studies was given in Table 3.

## 9. Host Restriction Factors in Bovines: Sensitivity of Influenza to Cellular/Serum Factors

Despite the growing host range of IAV over the last century, the rare incidence of influenza A in bovine species could be due to the presence of some host-dependent restriction factors in the bovine respiratory tract which could possibly hinder/interfere with influenza virus replication and further adaptation. The fact that influenza A and B viruses could replicate almost always in the in vitro bovine systems [145,155,162,163,185,186,187,188] but very rarely in vivo, suggests the presence of an interfering host factor in vivo. Previous research has shown that biological factors/fluids of ruminants possess anti-influenza activity and hence we speculate that the lack of sensitivity of bovine species to influenza viral pathogenesis could be attributed to some host factors, which could be physiological, cellular, or related immune factors.

### 9.1. Bovine Colostrum/Milk

Bovine milk can inactivate/interfere with the hemagglutinating property of influenza virus [189]. Bovine IgG present in the milk binds with viruses aiding phagocytosis. Similarly, the oligosaccharides in the milk can act as a decoy receptor preventing the binding of the influenza virus to the sialylated glycans on the cells [190,191]. Ruminant biological fluids/components such as bovine colostrum, amniotic fluid, mucoprotein from bovine submaxillary glands also could interfere with biological properties of influenza [192,193,194,195,196,197]. A consumable low-molecular-weight fraction (CLMWF) of immunoglobulin-depleted bovine colostrum whey, exhibited antibacterial (Streptococcus) and antiviral (influenza) immune defense in vivo, by aiding the maturation of the antigen presenting cells [198]. Oral administration of the bovine late colostrum has been found to have augmented the local and systemic cellular immunity and the activation of cellular immunity in mice by increasing the NK cell activity, together with high levels of IL-12 and IFN-γ [195].

### 9.2. Bovine Lactoferrin

Bovine lactoferrin (bLf) is an important protein with broad-spectrum anti-influenza activity [199,200,201,202]. Lactoferrin is a 76 kDa glycoprotein with a single polypeptide chain of 689 amino acid residues, present in the biological fluids and specific granules of polymorphonuclear leukocytes, which has a potential role in immunomodulation, iron absorption, and pathogen inhibition. Bovine lactoferrin demonstrated an inhibiting property against enveloped viruses [203]. Structurally, bLf is a protein with two symmetrical and globular lobes: C- and N-lobe, each with two sub-domains, I and II with an interdomain cleft that binds to an iron atom. C-lobe binds with the HA stem region which includes HA2 and some important amino acid residues of the HA1 region spanning the universal conserved epitope, which explains the broad-spectrum anti-influenza activity of the bLf C-lobe [203]. At the molecular level, bovine lactoferrin binds to the influenza virus HA, thus inhibiting H1N1 and H3N2 influenza viruses [199]. Pietrantoni et al. [200,201] demonstrated that bLf interferes with caspase 3 function and inhibits the nuclear export of the viral ribonucleoproteins to the cytoplasm. This function of bLf was maintained in its desialylated, deglycosylated, apo, and ion-saturated forms. Superti et al. [204] demonstrated that bovine lactoferrin interacts with HA at low pH to attain a stable conformation of the HA of IAV (H1N1), thereby inhibiting the fusion peptide activity [204]. Bovine lactoferrins were also known for its anti-infective, anti-cancer, and anti-inflammatory effects. Oral administration of the bovine lactoferrins reduced the lung consolidation score and leucocyte infiltration in the bronchoalveolar lavage fluid in mice [202,205].

### 9.3. Bovine Serum Inhibitors: Conglutinin, Collectin

The sensitivity of influenza viruses to periodate-resistant inhibitors in the normal bovine serum has been studied earlier [206]. Bovine sera, especially from fetal calf or normal calf, induced the formation of hemagglutination inhibitors upon treatment with potassium periodate, similar to the phenomenon observed with treated sera from human, rabbits, rats, guinea pig, horses, goat, chicken, and monkey. Only the mouse and hamster sera did not show any increase in the HI titer after periodate treatment [207]. Furthermore, a quantitative estimation of the non-specific HA inhibitors of influenza virus present in the sera of 27 species involving laboratory, domestic, and wild animals and birds demonstrated that sera of sheep, goats, and cattle belonged to a separate group, which is based on the physicochemical properties of antiviral inhibitors such as its sensitivity to heating, potassium periodate, trypsin, 2-Mercaptoethanol, and rivanol. This study also demonstrated that non-specific inhibitors present in bovine sera are heterogenous, i.e., both thermolabile and thermostable types [208]. The non-Ig inhibitors, also known as beta inhibitors in the normal bovine and mouse sera, are mannose-binding lectins [209,210] which bind to the carbohydrate moieties on the HA glycoprotein, blocking the receptor binding sites and thus inhibits the infectivity and hemagglutinating activity of the H1 and H3 influenza A viruses. These serum inhibitors in bovine serum resembled conglutinin, which is a Ca (2+) dependent N-acetylglucosamine and mannose-binding lectin and its hemagglutination inhibition property was abrogated by the polyclonal and monoclonal anticonglutinin antibodies [211,212]. These conglutinins can also act as opsonins for the phagocytosis of influenza A viruses [213]. Wakamiya et al. [214] isolated and characterized conglutinin and demonstrated that the beta-like inhibitor activity of bovine serum is attributed to conglutinin which prevents hemagglutination and neutralizes the virus infectivity. A recombinant bovine conglutinin demonstrated sugar binding, hemagglutination inhibition, conglutination, and neutralization activity against influenza A viruses, such as the native conglutinin [215]. Bovine serum proteins such as conglutinin, collectin-43 (CL-43) and collectin-46 (CL-46) are C-type lectins, of which conglutinin and CL-43 exhibited antiviral properties against influenza A and rotaviruses [216,217]. Conglutinin in the serum of the dairy cows is dependent on the season, breeding, stage of the reproductive cycle and infection [218].

### 9.4. Bovine Lung Factors

Aprotinin, a natural 58 amino acid protease inhibitor of bovine lung origin, already intended to use in humans for pancreatitis and hemorrhage also possesses the potential to suppress the cleavage of the pandemic H1N1 influenza virus in different host systems such as human tracheobronchial epithelium, human intestinal Caco-2 cells and chicken embryonated eggs [219]. Aprotinin inhibits serine proteases needed for the cleavage of HA and thereby suppressing influenza activity [220]. Similarly, bovine pulmonary Surfactant obtained by the endotracheal lavage of bovine lungs is an efficacious adjuvant for intranasal vaccination and is widely used for acute respiratory distress syndrome in newborns. A synthetic analog for this compound, called SF-10 is available to avoid the risk of Bovine spongiform encephalopathy (BSE). Intranasal application of HA adjuvanted with SF-10 in mice stimulated higher levels of anti-HA-specific secretory IgA (s-IgA) in nasal-wash and serum IgG than induced by HA-poly (I:C), a mucosal vaccine used for protection [221]. SF-10 is an excellent adjuvant choice for commercial vaccine production as it could induce both mucosal and systemic immunity, with balanced Th1-/Th2-type cytokine responses with the production of anti-HA-specific IgG1 and IgG2a without any anti-HA IgE along with IFN-γ- and IL-4-producing lymphocytes in the nasal cavity [221].

### 9.5. Bovine Antivirals

The role of antiviral factors in the influenza-related innate immune responses have been widely studied as early as the 1960s and found a direct correlation of antiviral protective effect of the interferons on the infected cells [222]. Recombinant bovine interferons (α and γ) were used to study the influenza replication and found that the interferons -α and not -γ, induced the formation of Mx proteins which possess antiviral activity [223]. Mx proteins are dynamin-like large guanosine triphosphatases (GTPases). Different orthologs/paralogs of Mx proteins could be found in either nucleus or cytoplasm, and possess unique antiviral activity depending on the species origin [224]. Anti-influenza activity of the human and bovine chimeric Mx proteins was studied by substituting the GTPase effector domain (GED) of the human Mx protein with bovine and vice versa, and demonstrated that bovine Mx1 proteins exhibited an increased activity against the influenza virus particularly the motifs located in its N-terminal portion is responsible for the interaction with the cellular and viral factors [225]. Table 4. describes the studies conducted in the bovines to detect the host-specific restriction factors against the influenza disease pathogenesis in a chronological manner. Taken together, these studies on the species-specific antiviral factors in bovines indicated that these factors possess anti-influenza activity. Further studies are needed to confirm their causal association and to identify more of such potential host-derived restriction factors.

## 10. Influenza Vaccine Studies in Bovines and Use of Bovine Viral Vectors

In the late 20th century, a vaccination study using killed vaccines of foot and mouth disease (FMD) and influenza was conducted in two-month-old calves and lambs. Interestingly, both these viruses exist in multiple serotypes. Formalin-inactivated allantoic viral cultures of A/PR/8/34 (H1N1) were used as a vaccine. In case of influenza, 100% of animals seroconverted against the homologous serotype influenza (A/PR/8/34) and 18.7% of animals seroconverted against each of the heterologous serotypes A/Leningrad/360/86 (H3N2) and A/Shanghai/11/87 (H3N2)} in calves. Similarly, in lambs, 100% seroconversion was observed against homologous serotype influenza (A/PR/8/34) and 10% and 17.5% seroconverted against the heterologous serotypes A/Leningrad and A/Shanghai, respectively. Only 1/32 and 1/40 responded to both heterologous serotypes in calves and sheep respectively [221].

Bovine adenovirus/adenoviral vector was used to replace the human adenovector to circumvent the pre-existing vector immunity against the human adenovectors in vaccine production. Administration of bovine adenovector (BAd) subtype 3 vectored H5-HA vaccine in human adeno (HAd) serotype 5 primed mice successfully eluded the high levels of pre-existing human adeno neutralizing antibodies and elicited HA-specific humoral and cell-mediated immune (CMI) responses. BAd vector immunization of naïve or adenovirus primed mice ensured full protection from a potentially lethal challenge with A/Hong Kong/483/97 in mice, which ensures its utility in influenza and as an alternative/supplement to other human adenovirus vectored vaccines [232,233]. Influenza viral vectors, particularly of subtypes H5N1 and H1N1 were used for veterinary vaccine applications. Effective protective efficacy was observed upon vaccination of influenza viral vector-based Brucella vaccine in cattle and small ruminants [234,235].

## 11. Summary

Here, we conducted a comprehensive review of the literature available on the past influenza cases/studies occurring globally in ruminant (bovine, caprine, ovine) population, and have summarized the overall influenza A prevalence in bovines. In this review, we have discussed the host range of the four types of influenza, emphasizing the susceptibility/utility of bovine in vivo and in vitro models to influenza A studies over recent years. Even though natural cases of influenza occurred in bovines causing influenza-like respiratory disease with bronchopneumonia, epizootic cough, nasal discharge, lacrimation, or other extrapulmonary signs such as milk drop, only a few cases culminated in successful virus isolation. Cattle-origin IAV strains were isolated during the early 1970s, around the time when Hongkong/1968 human IAV strains (H3N2) were prevalent. Although the relatedness of HA glycoprotein of these cattle IAV strains to human H2 and H3 prototypes was reported, sufficient data/characterization studies were lacking to support the extent of genetic relatedness. Pigs, which were domesticated by humans 1500 years after cattle, are naturally susceptible to all four influenza types and are excellent mixing vessels of influenza. Hence, the refractory nature of bovines against influenza A could be due to species-specific host-associated interference as discussed before. Compared to IAV and IBV (eight segmented genomes), bovines are naturally susceptible to IDV, and lately to ICV (seven segmented genomes) as indicated by the seroprevalence studies and isolation of complete viral genomes, thus contributing significantly to the bovine respiratory disease along with other bacterial/viral pathogens. The transboundary occurrence of influenza D in bovines, compounded with its extraordinary thermal and pH stability [236] compared to other influenza types, demands further studies to study the pathobiological aspects of this virus and its predisposition in bovine species. The fact that bovines harbor some natural predisposing factors, amenable for the tissue tropism and pathogenesis of ICV and IDV, while detrimental to IAV and IBV, would make them a suitable model to delineate influenza type-specific host–pathogen interactions, and further studies are needed to address this differential disease pathogenesis at the cellular and molecular level.

## Figures and Tables

**Table 1:** Natural and experimental cases of influenza A associated with ruminant species.

Description	Year	Ref.
**Natural infection**		
Influenza outbreak in Japan	1951	[76]
*Incidence, level of influenza, and adeno virus antibodies in domestic animal species	1969	[25]
*Antigenic characteristics of influenza viruses from domestic animals and birds (USSR)	1973	[89]
*Influenza outbreak in cattle	1973	[90]
Isolation of influenza A strains from cattle	1973	[46]
*Study of cattle influenza	1976	[92]
*Influenza in cattle	1977	[91]
Hong Kong influenza A strains in calves	1977	[48]
*Pathological anatomical examination of the lung from bovine influenza	1977	[94]
*Influenza of cattle	1978	[93]
Unexplained sporadic milk drop in cows	1997	[95]
Bovine influenza	1998	[97]
Influenza A in dairy cows with sporadic milk syndrome	1999	[98]
Evidence of antibodies in sera/nasal samples against human influenza viruses from 17 outbreaks of respiratory disease with milk drop syndrome and diarrhea in cattle in 1998–1999	2002	[99]
Wild animals as a reservoir for different bacterial and viral diseases including avian influenza	2002	[102]
**Experimental infection**		
*Experimental infection of bovines with human influenza virus	1954	[26]
*Experimental inoculation of calves with influenza virus A/csf/Udmurtiia/116/73	1977	[27]
Experimental infection of influenza in yak and presence of H3N2 antibodies	1974	[103]
Recombinant vaccinia virus expressing HA in cattle, sheep, and poultry	1986	[104]
Intranasal inoculation of calves with live swine influenza virus	1987	[105]
Experimental inoculation of a cat derived highly pathogenic avian influenza virus in calves	2008	[106]
Experimental inoculation of highly pathogenic avian influenza virus H5N1 in cattle egrets	2011	[107]

Articles in non-English languages*.

**Table 2:** Serosurveillance studies of influenza A associated with ruminant species in the past.

Description	Year	Ref.
Influenza A and B specific antibodies in domestic and wild animals	1965	[118]
Serological study in Ottawa based on immunoprecipitation test found influenza A antibodies in sheep and goat among 14 species tested	1975	[119]
Serosurveillance of swine H1N1 in cattle and swine in Great Britain	1978	[120]
Serological screening of influenza B and C in cattle, horses and other animals in Japan	1978	[56]
Serological evidence of influenza A in cattle in Japan	1978	[111]
Influenza-specific antibodies not detected in indigenous and non-indigenous sheep breeds of Northern Ireland	1984	[121]
Influenza in ruminants: a review with information regarding viruses isolated from the cattle	1984	[47]
Single radial hemolysis to measure influenza antibody in cattle serum	1986	[123]
Swine influenza virus as a component in the respiratory disease complex in calves	1986	[122]
Presence of influenza A specific antibodies in cattle with respiratory disease and reduced milk yield	1998	[96]
Vaccination study of foot and mouth disease and influenza in cattle and sheep	1998	[125]
Influenza A antibodies associated with an acute reduction in milk yield in cattle in Britain	2008	[100]
Serosurveillance study of avian influenza H5N1 in cattle, buffaloes, sheep, goat, and other animals in Egypt	2013	[124]

**Table 3:** Utility of bovine cell/tissue cultures for influenza A studies in the past.

Description	Year	Ref.
Studies on the cellular enzymes and their role in the cytopathic effect of influenza in cell cultures	1967	[181]
Replication of WSN influenza virus in high titers in MDBK cell line at high and low multiplicity of infection	1969	[144]
*Adaptation of the influenza virus in calf kidney cell cultures	1969	[182]
Study of infective and incomplete influenza virions grown in MDBK and HeLa cells	1970	[145]
Calf serum suppressed plaque formation of many influenza virus strains in different cell lines	1970	[183]
Influenza B virus propagation in bovine fetal kidney cell cultures: incomplete virus formation	1970	[148]
Influenza B virus forms plaque in primary calf kidney cells	1971	[156]
Interaction of swine influenza and bovine mycoplasma in bovine tracheal cultures	1971	[177]
*Influenza virus adaptation (A2 (Hong Kong) 68) in calf kidney cell cultures	1972	[184]
Use of bovine kidney cells for the propagation of *ts* recombinant viruses	1972	[173]
Use of primary bovine kidney cells at 25 °C for growing low temperature adapted vaccine virus	1973	[174]
Influenza virions grown in Madin–Darby Bovine Kidney (MDBK) cells without calf serum have more uncleaved HA especially in the early phase indicating that HA cleavage is both host cell and strain dependent	1973	[166]
Influenza virus grown in MDBK cells in the presence of medium containing 2% calf serum caused cleavage of HA polypeptide, to HA1 and HA2 unlike serum-free medium due to the plasminogen component in the sera	1973	[167]
Use of bovine kidney and trachea organ cultures for influenza for studying the virus reactogenic and immunogenic properties	1975	[178]
Study of the polypeptide composition of incomplete influenza viruses propagated in MDBK cells	1975	[185]
Use of lung and trachea organ cultures from bovine and other species for the influenza studies	1976	[176]
Use of bovine embryo kidney cells, roller cultures for high titer influenza virus propagation	1977	[157]

Articles in non-English languages*.

**Table 4:** Summary of major bovine host antiviral factors and their interactions with influenza.

Description	Year	Ref.
Inhibition of influenza virus hemagglutination by cow’s milk	1949	[189]
*Inhibition of influenza virus hemagglutination by bovine amniotic fluid factor	1954	[193]
Mucoprotein from bovine submaxillary glands with restricted hemagglutination inhibition activity against influenza virus	1955	[192]
Influenza virus inhibitor in human and cow milk	1960	[226]
*Beta inhibitors in bovine serum	1963	[227]
Use of sera from different species of animals to study periodate induced hemagglutination inhibitor	1968	[207]
Influenza A2/Hong Kong strains were sensitive to periodate-resistant inhibitors in normal bovine serum	1971	[206]
*Serum inhibitors of hemagglutination	1972	[228]
Serum inhibitors of hemagglutination of A2/Hong Kong strains	1972	[229]
*Characterization of non-specific inhibitors of hemagglutination of influenza A virus in the sera of different species of animals and birds	1977	[208]
*Serum inhibitors of cattle and influenza virus persistence in Madin–Darby Swine Kidney (MDSK) cells	1980	[230]
Conglutinin as bovine serum beta inhibitor of influenza virus hemagglutination and infectivity of H1 and H3 subtypes	1992	[212]
Isolation of influenza virus inhibitor, conglutinin	1992	[214]
Opsonizing activity of conglutinin against influenza A virus	1993	[213]
Recombinant bovine conglutinin deficient in N-terminal and collagenous domains and its activity against influenza virus	1996	[211]
Bovine collectins and its antiviral activity against rota virus	1998	[216]
CL-43, bovine serum collectin, and its antiviral activity by inhibiting the hemagglutination activity of influenza A virus	2002	[231]
Bovine collectins_Conglutinin, CL-43, and CL-46	2006	[218]
Recombinant trimeric neck and carbohydrate recognition domains (NCRD) of bovine conglutinin and CL-46 demonstrated a higher level of intrinsic antiviral activity against influenza A virus	2010	[217]
Aprotinin, a natural polypeptide of bovine lung origin can inhibit the HA cleavage of pdmH1N1 and its replication in different host systems	2011	[219]
Bovine colostrum can enhance natural killer cell activity and thus boosts the immune response against influenza in a mouse model	2014	[196]
Peptide inhibitors derived from lactoferrin C-lobe possess broad anti-influenza activity and prevented influenza hemagglutination and infection	2012, 2017	[199,203]
Bovine lactoferrin interferes with the fusion of HA glycoprotein and thus inhibits influenza A infection	2019	[204]

Articles in non-English languages*.
